# Jasmonic acid signaling and glutathione coordinate plant recovery from high light stress

**DOI:** 10.1093/plphys/kiaf143

**Published:** 2025-04-10

**Authors:** Mehmet Kılıç, Peter J Gollan, Eva-Mari Aro, Eevi Rintamäki

**Affiliations:** Molecular Plant Biology, Department of Life Technologies, University of Turku, Turku 20014, Finland; Molecular Plant Biology, Department of Life Technologies, University of Turku, Turku 20014, Finland; Molecular Plant Biology, Department of Life Technologies, University of Turku, Turku 20014, Finland; Molecular Plant Biology, Department of Life Technologies, University of Turku, Turku 20014, Finland

## Abstract

High light (HL)-induced chloroplast retrograde signaling originates from the photosynthetic apparatus and regulates nuclear gene expression to enhance photoprotection and coordination of cell metabolism. Here, we analyzed the transcript profiles and accumulation of ROS, stress hormones, and small molecule antioxidants to investigate the signaling mechanisms operating under HL stress, particularly during plant recovery under growth light condition. Exposure of Arabidopsis (*Arabidopsis thaliana*) rosettes to HL for 15 min induced several ^1^O_2_- and H_2_O_2_-responsive genes and accumulation of an oxidized form of glutathione, the hallmarks of oxidative stress in cells. Prolonged exposure to HL resulted in accumulation of transcripts encoding oxylipin biosynthesis enzymes, leading to accumulation of 12-oxo-phytodienoic acid and jasmonic acid. However, the expression of several jasmonic acid-responsive genes, already induced by HL, peaked during the recovery, together with accumulation of jasmonic acid and reduced glutathione and ascorbate, highlighting the critical role of jasmonic acid signaling in restoring chloroplast redox balance following HL stress. The involvement of jasmonic acid signaling in recovery-sustained gene expression was further confirmed via experiments with jasmonic acid receptor mutants. HL exposure of only 2 min was sufficient to induce some recovery-sustained genes, indicating the rapid response of plants to changing light conditions. We propose that ROS production at HL induces the signaling cascade for early oxylipin biosynthesis and 12-oxo-phytodienoic acid accumulation, while increased accumulation of jasmonic acid in the recovery phase activates genes that fully restore the glutathione metabolism, ultimately allowing recovery from short-term HL stress.

## Teaser

ROS production at high light induces oxylipin biosynthesis, while jasmonic acid acts as a molecular memory of the stress and activates gene expression promoting recovery from high light stress.

## Introduction

Photosynthesis plays a crucial role in sensing environmental cues and relaying signals for regulation of plant acclimation (Walters 2005; Gollan et al. 2015; Morales and Kaiser 2020). Photosynthetic machinery converts environmental cues into biochemical messengers that adjust the expression of genes in both the cell nucleus and plastids. This acclimation response helps the organism cope with stresses and acclimate to alternating environments. Photosynthetic light reactions occurring in photosystem (PS) II and PSI together with their light-harvesting complexes, collect and transform light energy into chemical energy to fuel the assimilation of atmospheric CO_2_ into organic energy-rich compounds. Environmental stress, such as high light (HL), can disrupt the electron flow, leading to production of reactive oxygen species (ROS) and reactive electrophile species (RES) (Farmer and Davoine 2007; Khorobrykh et al. 2020; Fitzpatrick et al. 2022) which, if not controlled, are hazardous to biological molecules. In addition to cellular damage, ROS and RES also initiate signals to protect and acclimate the photosynthetic machinery from deleterious effects of redox changes in the chloroplast.

Oxylipins are important redox components regulating growth and stress responses in plants (recent review, see Knieper et al. 2023). ROS induces the synthesis of oxylipins, including 12-oxo-phytodienoic acid (OPDA) and jasmonic acid (JA). They are derivatives of oxygenated α-linolenic acid released from plastid membranes by lipases, and are synthesized in an enzymatic pathway (Farmer and Davoine 2007; Wasternack and Song 2017; Knieper et al. 2023). OPDA synthesis starts with the oxygenation of α-linolenic acid by 13-LIPO-OXYGENASE producing 13-hydroperoxy-octadecatrienoic acid (13-HPOT), which is then processed to OPDA by ALLENE OXIDASE SYNTHASE (AOS) and ALLENE OXIDASE CYCLASE (Schaller and Stintzi 2009; Wasternack and Song 2017). OPDA is transported from plastids to peroxisomes to synthesize JA by 12-OXOPHYTODIENOATE REDUCTASE 3 followed by 3 cycles of β-oxidation catalyzed by ACYL-COA OXIDASE (Schaller and Stintzi 2009; Wasternack and Song 2017). *JAR1* gene is required for biological activation of JA via conjugation with isoleucine (JA-Ile) (Suza and Staswick 2008; Wasternack and Song 2017).

Both OPDA and JA function as signaling molecules modifying gene expression in the nucleus (Chini et al. 2007; Wasternack and Song 2017). Oxylipins that contain α, β-unsaturated carbonyl bonds, such as OPDA, are categorized as RES due to their inherent reactivity with thiol groups of cellular proteins (Alméras et al. 2003; Farmer and Davoine 2007). OPDA and JA were observed to take part in antioxidant defense response by inducing antioxidant gene expression (Sasaki-Sekimoto et al. 2005; Gollan and Aro 2020). JA is sensed by its receptor CORONATINE INSENSITIVE 1 (COI1) (Wasternack and Song 2017) that is a subunit of the E3 ubiquitin protein ligase (SKP1-Cullin-F-box, SCF^COI1^) (Yan et al. 2013; Wasternack and Song 2017). SCF^COI1^ labels proteins with ubiquitin to induce the proteolytic degradation (Chini et al. 2007; Yan et al. 2013). JA-Ile binds to the SCF^COI1^ complex and facilitates the ubiquitination and degradation of the JAZ transcriptional repressors, allowing transcription factors to bind to JA-responsive genes to initiate transcription (Chini et al. 2007; Dombrecht et al. 2007). Via this induction mechanism, JA signaling is involved in regulation of CO_2_ fixation, antioxidant metabolism, degradation of chlorophylls, reallocation of resources from growth toward defense systems, and senescence (Sasaki-Sekimoto et al. 2005; Shan et al. 2011; Park et al. 2013; Zhang et al. 2020).

Cells harbor both enzymatic and non-enzymatic ROS scavenging systems to protect against excessive damage by ROS. Enzymes, such as SUPEROXIDE DISMUTASEs, CATALASEs, ASCORBATE PEROXIDASEs, GLUTATHIONE PEROXIDASE-LIKE proteins, and PEROXIREDOXINs directly detoxify ROS, whereas other enzymes such as THIOREDOXINs and GLUTAREDOXINs re-reduce proteins oxidized by ROS. Nonenzymatic ROS scavenging system includes molecular antioxidants such as ascorbate, tocopherols, and glutathione, which act as electron donors to neutralize ROS. Glutathione is involved in scavenging of both ROS and RES (Dixon and Edwards 2009; Foyer and Noctor 2011; Ito and Ohkama-Ohtsu 2023), thereby preserving the cellular integrity (see the recent reviews by Dorion et al. 2021; Noctor et al. 2024). Oxidized GSH (GSSG) is reduced back to GSH by GLUTATHIONE REDUCTASE (GR) and NADPH.

Short-term HL treatments lasting from seconds to few minutes induce photosynthetic regulatory mechanisms in chloroplasts, such as non-photochemical quenching, photosynthetic control, and changes in the activation status of key enzymes in Calvin-Benson-Bassham cycle, helping the plant to cope under fluctuating light conditions (Niyogi and Truong 2013; Ustynyuk and Tikhonov 2018; Nikkanen and Rintamäki 2019). Longer HL treatments, ranging from minutes to hours, enhance metabolic processes that increase tolerance against oxidative stresses (Dietz 2015; Gollan and Aro 2020). Induction of protective gene expression and antioxidant metabolism has been extensively studied in HL-treated plants (Hernández et al. 2004; Chan et al. 2016; Balfagón et al. 2019; Huang et al. 2019; Alvarez-Fernandez et al. 2021; Balcke et al. 2024), while far less is known about their fate after transferring the plants back to growth light (GL) conditions for recovery (R) (Crisp et al. 2017). In this study, we focused on the R phase after HL treatment and analyzed the changes in global gene expression together with the stress hormone and antioxidant levels in leaves exposed to short-term HL stress and during the subsequent R from stress at GL. We show that JA is an important mediator of nuclear gene expression in leaves during R from HL stress and that GSH helps to eliminate ROS and rebalance the redox state of the cell when HL illumination is terminated.

## Results

**Figure 1.**
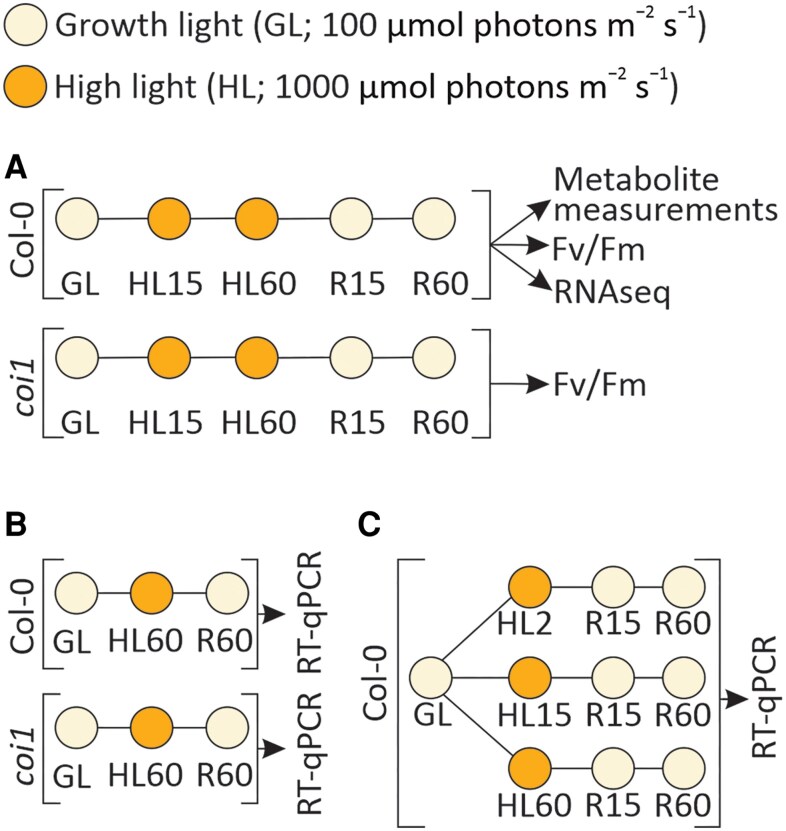
Experimental design to investigate the effect of high light (HL) and subsequent recovery (R) on gene expression and metabolism in Arabidopsis leaves. **A)** Wild type Col-0 plants and *coi1* mutants grown under growth light (GL, 100 μmol photons m^−2^ s^−1^) were exposed to HL (1000 μmol photons m^−2^ s^−1^) for 15 (HL15) and 60 min (HL60). For recovery, plants exposed to HL for 60 min were returned to GL conditions and allowed to recover for 15 min (R15) and 60 min (R60). WT plants were used for metabolite and RNAseq analysis, and WT and *coi1* mutant plants were used for determination of photosynthetic efficiency of PSII (Fv/Fm). **B)** GL grown Col-0 plants and *coi-1* mutants were exposed to HL for 60 min (HL60) and returned to GL to recover for 60 min (R60). The treated plants were used for analysis of jasmonic acid (JA)-responsive gene expression with reverse transcription quantitative PCR (RT-qPCR). **C)** GL grown Col-0 plants were exposed to HL for 2 (HL2), 15 (HL15) and 60 min (HL60), and returned to GL to recover for 15 (R15) and 60 min (R60). The treated plants were used for time dependent analysis of JA-responsive gene expression with RT-qPCR.

The rosettes of Arabidopsis (*Arabidopsis thaliana*) Columbia (Col-0) ecotype were grown at 100 *μ*mol photons m^−2^ s^−1^ (GL) for 6 wk, followed by exposure to 1000 μmol photons m^−2^ s^−1^ for 2, 15 min (HL15), or 60 min (HL60), while subsequent recovery from HL treatments involved illumination under GL for 15 min (R15) or 60 min (R60). Leaf number 7 of Arabidopsis rosettes was used for metabolite and gene expression analyses (Fig. 1).

**Figure 2.**
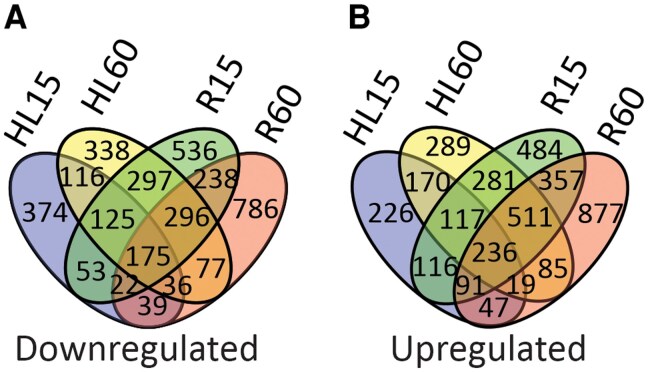
Differentially expressed genes in high light (HL) and during recovery (R) in comparison to growth light (GL). **A)** Number of downregulated genes in treated leaves in comparison to GL. **B)** Number of upregulated genes in treated leaves in comparison to GL. HL treatment was performed by exposing plants to HL for 15 (HL15) and 60 min (HL60), while recovery was performed by transferring HL60 samples to GL to recover for 15 (R15) and 60 (R60) min after HL treatment. Only the genes with statistically significant changes with fold change (FC) of −1 > log2 FC > 1 (*P* < 0.05) are included in Venn diagrams.

Global gene expression analysis was performed after the HL and subsequent R treatment for 15 and 60 min, and the results were expressed relative to the GL control without any HL treatment. Distinct patterns of differential gene expression were identified in each phase. A total of 7,414 differentially expressed genes (DEGs) were detected in the experiment compared to the GL control (Fig. 2). The number of DEGs was substantially higher during R compared to the previous HL treatment: 44% of the DEGs were specific for the R period compared to 20% of DEGs for the HL period, (Fig. 2). The highest number of DEGs was observed at R60, while the lowest number was recorded at HL15 (Fig. 2). This indicated that considerable reprogramming and adjustment of gene expression occur in the cell during R phases after HL treatment.

### JA, OPDA, and GSH are involved in regulation of gene expression during recovery from HL treatment

We used the DEGs to identify, at the transcript level, the various biological processes that were affected during the HL and R phases of the experiment. Gene Ontology (GO) terms related to ROS responses were particularly enriched in genes upregulated during the HL treatment (Supplementary Table S1). Transcripts of glutathione metabolism and JA signaling, on the other hand, accumulated during HL60 as well as R15 and R60 (Supplementary Table S1). GO terms related to abscisic acid (ABA) signaling were moderately upregulated in HL15, whereas GO terms related to salicylic acid (SA) signaling were upregulated in both HL and R, especially in HL15 and R15 (Supplementary Table S1).

**Figure 3.**
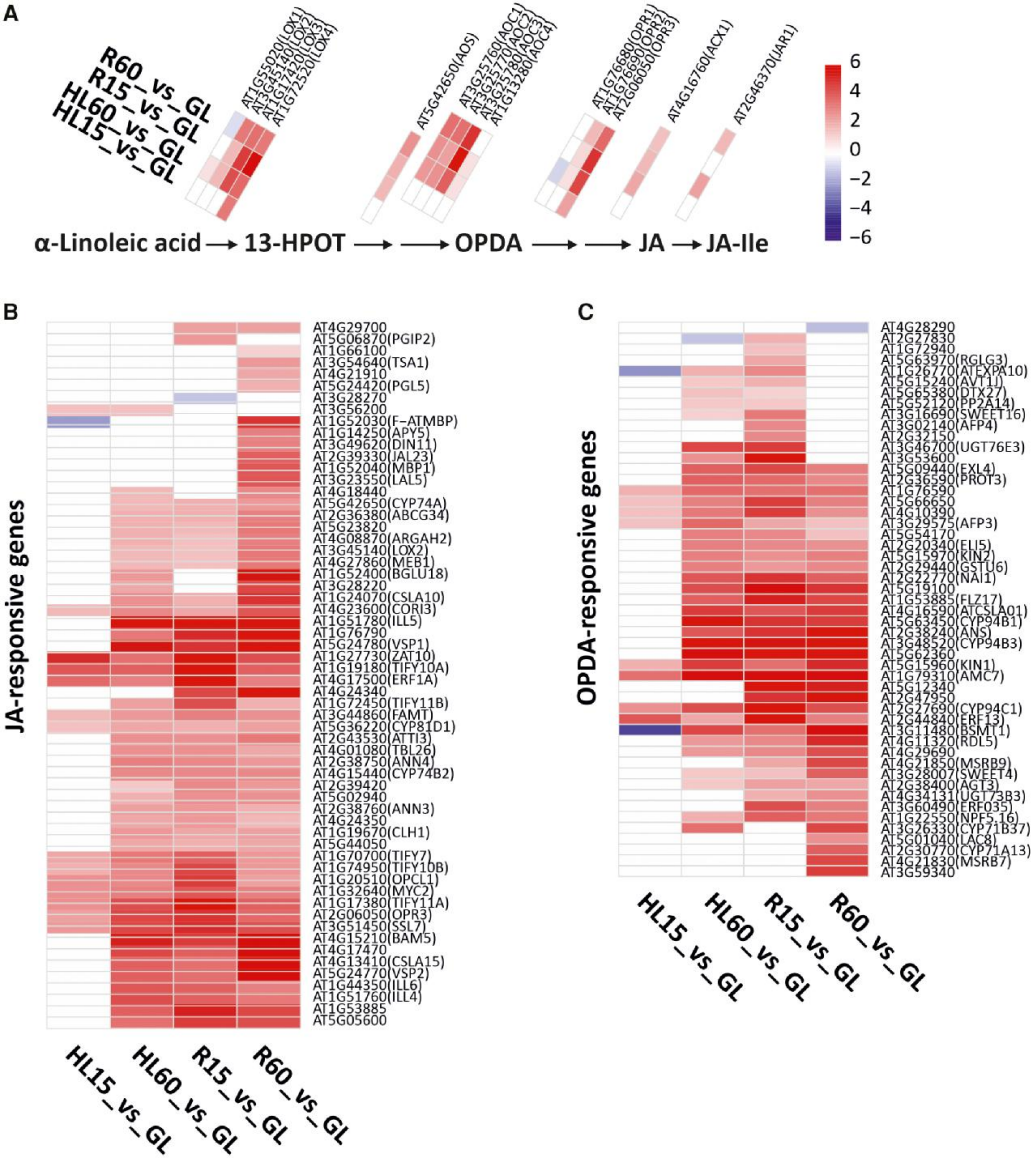
Differential expression of oxylipin-responsive genes in leaves treated in high light (HL) and in recovery (R) at growth light (GL) in comparison to control GL leaves. HL treatment was performed by exposing plants to HL for 15 (HL15) and 60 min (HL60), while recovery was performed by transferring HL60 samples to GL to recover for 15 (R15) and 60 (R60) min. **A)** Differential expression of 12-oxo-phytodienoic acid (OPDA) and jasmonic acid (JA) synthesis genes. Gene names are listed in the text. 13-hydroperoxy-octadecatrienoic acid (13-HPOT); conjugation of JA with isoleucine (JA-Ile). **B)** Differential expression of JA-responsive genes. **C)** Differential expression of OPDA-responsive genes. The genes indicated in **B** and **C)** were previously reported to be upregulated in response to OPDA and JA treatments in (Gollan and Aro 2020). Red and blue color scale (log2-fold change) shows the degree of upregulation and downregulation of the genes, respectively. Only the statistically significant log2-fold changes (*P* < 0.05) in gene expression in comparison to GL are shown in the figure. Non-significant values were replaced by 0 in the heat map.

Since transcripts of hormone and oxidative stress-related biological processes were found to be enriched in the GO analysis (Supplementary Table S1), we next analyzed our gene expression data with respect to known responses induced by metabolites such as singlet oxygen (^1^O_2_), hydrogen peroxide (H_2_O_2_), ABA, OPDA, JA and SA (Op Den Camp et al. 2003; Xin et al. 2005; Gollan and Aro 2020; Zhang et al. 2020). Most of the OPDA and JA biosynthesis genes were upregulated in HL60 and remained at a high level or even increased during R (Fig. 3A). A similar trend was also observed for other JA- and OPDA-responsive genes (Fig. 3, B and C).

The expression of ^1^O_2_-responsive genes peaked at R15 (Supplementary Fig. S1A), whereas the expression of H_2_O_2_-responsive genes increased with HL treatment and decreased during the R phase (Supplementary Fig. S1B). About half of the ABA-responsive genes showed increased expression in HL60 and R15, whereas the expression of SA-responsive genes did not change substantially in HL or during R (Supplementary Fig. S2).

**Figure 4.**
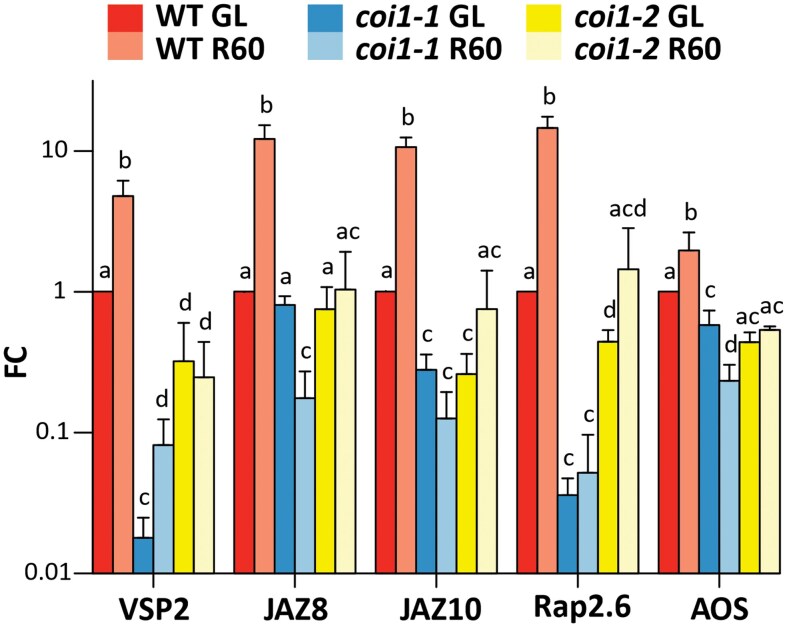
Relative expression of selected recovery-sustained genes in the wild type (WT) and *coi1* mutants. Reverse transcription quantitative PCR (RT-qPCR) assay was performed from leaves illuminated at growth light (WT GL; *coi1-1* GL; *coi1-2* GL) and from plants treated with high light (HL) for 60 min and then returned to GL for 60 min to recover from HL stress (WT R60; *coi1-1* R60; *coi1-2* R60). The following R-sustained genes were analyzed: *VSP2*, *JAZ8*, *JAZ10*, *Rap2.6* and *AOS*. The fold change (FC) of gene expression, normalized to WT GL values, is expressed on a logarithmic scale. Values represent the mean ± SD of 3 independent samples. Statistical analysis (ANOVA test, Tukey-HSD, *P* < 0.05) was done before transformation of the values to logarithmic scale. Letters indicate significant differences between the treatments.

JA-responsive genes that were activated at HL60, but their transcripts strongly increased during the R phase (Fig. 3), are called R-sustained genes hereafter. To confirm that JA is indeed involved in induction of R-sustained genes, we further tested the expression of a specific set of R-sustained marker genes (*VSP2*, *JAZ8*, *JAZ10, Rap2.6*, and *AOS*, Supplementary Table S2) in mutants lacking the JA receptor COI1 (*coi1-1*, *coi1-2*) by reverse transcription quantitative PCR (RT-qPCR) analysis. In this experiment, the leaves of wild type (WT) and the 2 *coi1* mutants were collected from the GL and after the exposure of leaves first to HL treatment for 60 min and then for 60 min to GL for recovery (R60) (Fig. 1B). Under initial GL conditions, the expression of selected genes was strongly (*VSP2*, *JAZ10* and *Rap2.6*) or slightly (*AOS*) lower in both *coi1* mutants compared to WT (Fig. 4). After HL treatment for 60 min followed by R for 60 min, the marker genes were significantly upregulated in WT, but not in the *coi1* mutants (Fig. 4), providing strong evidence that the JA signaling indeed initiates the expression of these genes during the R phase.

### Short HL exposure is sufficient to enhance the expression of JA-responsive recovery genes

**Figure 5.**
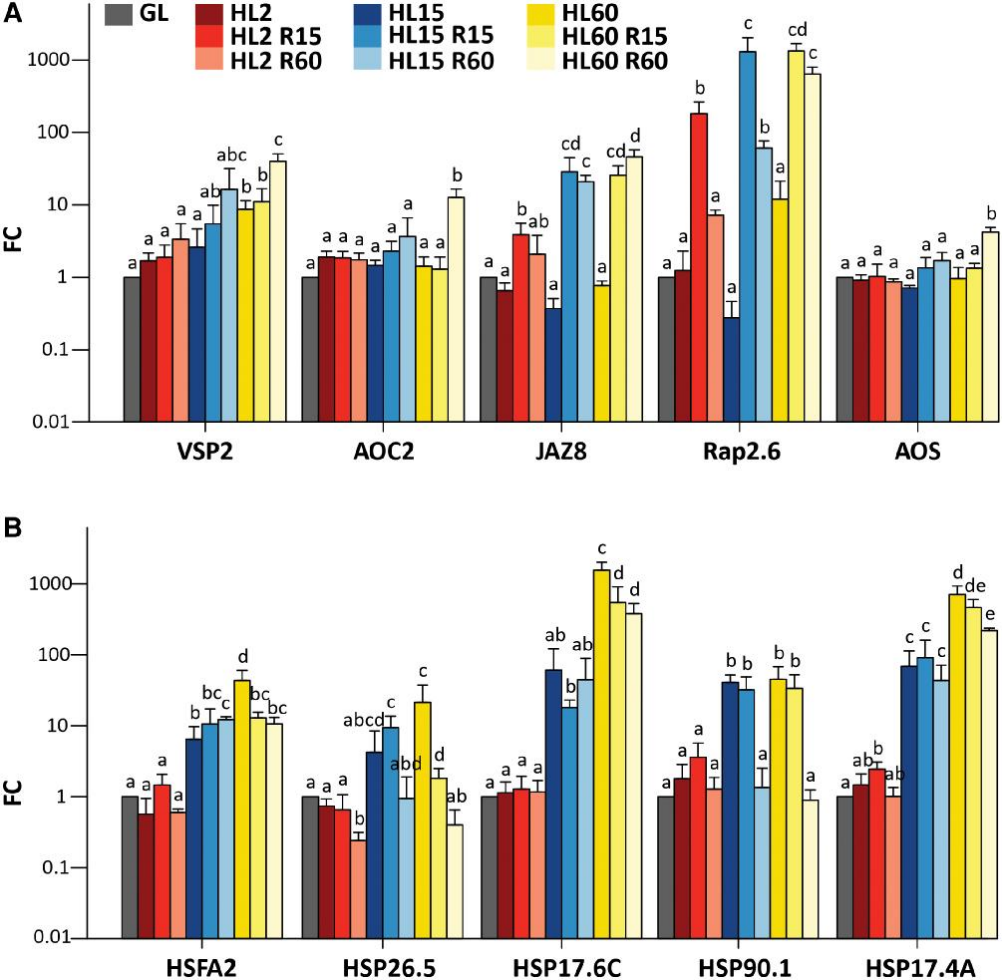
Relative expression of selected recovery-sustained genes and *HEAT SHOCK PROTEIN* (*HSP*) genes in wild type Arabidopsis leaves exposed to different durations of high light (HL) treatment followed by a recovery (R) period in growth light (GL). Reverse transcription quantitative PCR (RT-qPCR) measurements were performed on leaves taken from plants before HL treatment (GL) and after exposure to HL for 2 min (HL2), 15 min (HL15), or 60 min (HL60). After HL treatment, the leaves were returned to GL and the recovery from HL stress was monitored for 15 min (HL2 R15; HL15 R15; HL60 R15) or 60 min (HL2 R60; HL15 R60; HL60 R60). **A)** The following R-sustained genes were analyzed: *VSP2*, *AOC2*, *JAZ8*, *Rap2.6*, and *AOS*. **B)** The following HL-induced *HSP* genes were analyzed: *HSFA32*, *HSP26.5*, *HSP17.6C*, *HSP90.1*, and *HSP17.4A*. The fold change (FC) of gene expression, normalized to GL values, is expressed on a logarithmic scale. Values represent the mean ± SD of 4 independent samples. Statistical analysis (ANOVA test, Tukey-HSD, *P* < 0.05) was done before transformation of the values to logarithmic scale. Letters indicate significant differences between the treatments.

We then investigated the duration of the HL pretreatment required to enhance the expression of R-sustained marker genes in Arabidopsis leaves during the subsequent recovery in GL (Fig. 1C). For this analysis, the expression of specific R-sustained marker genes (*JAZ8*, *Rap2.6*, *VSP2*, *AOC2*, *AOS*) was determined by RT-qPCR (Fig. 5A). The expression of these genes remained largely unchanged during the HL treatment compared to GL, but a clear upregulation was observed during the R phase. Even a very short HL pretreatment of 2 min was sufficient to induce the upregulation of *JAZ8* and *Rap2.6* genes during the subsequent R phase, whereas *VSP2*, *AOC2* and *AOS* genes required a longer HL pretreatment of 60 min to be upregulated in the R phase (Fig. 5A). On the contrary, the expression level of selected *HSP* genes, known to be induced by HL (Supplementary Table S2), behaved differently. The expression of these genes already increased during HL but remained stable or decreased during R phase (Fig. 5B).

### Changes in leaf hormone and antioxidant contents during HL and recovery phase

**Figure 6.**
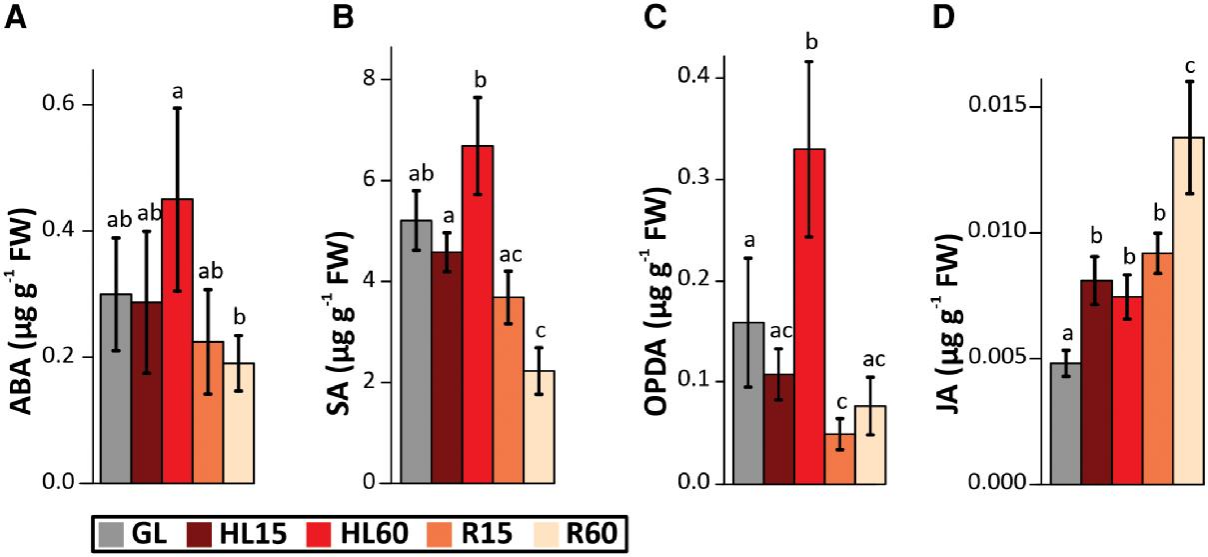
Concentration of stress hormones in leaves exposed to high light (HL) and subsequently transferred to recovery (R) conditions at growth light (GL). Measurements were made on leaves taken from plants before HL treatment (GL), after 15 min (HL15) and 60 min (HL60) of HL exposure, and during the R at GL for 15 min (R15) and 60 min (R60) after 60 min of HL treatment. **A)** Abscisic acid (ABA). **B)** Salicylic acid (SA). **C)** 12-oxo-phytodienoic acid (OPDA). **D)** Jasmonic acid (JA). The concentrations were expressed as μg in g of leaf fresh weight (FW). Values represent the mean ± SD of 4 independent samples. Letters indicate significant differences between the treatments (ANOVA, Tukey-HSD, *P* < 0.05).

**Figure 7.**
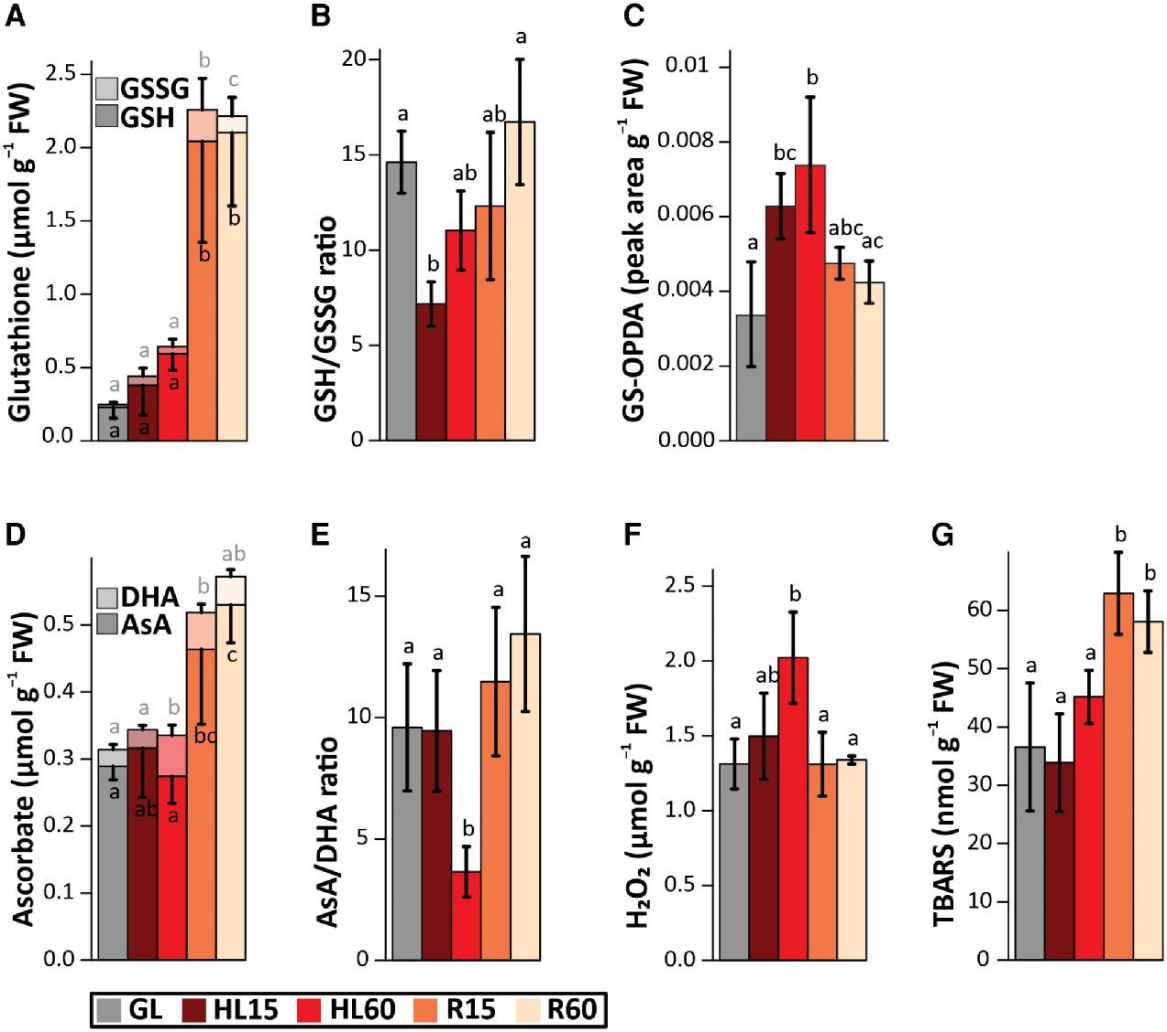
Concentration of antioxidants, hydrogen peroxide (H_2_O_2_), and thiobarbituric acid reactive substances (TBARS) in leaves exposed to high light (HL) and during subsequent recovery (R) at growth light (GL). Measurements were performed on leaves taken from plants before HL treatment (GL), after 15 min (HL15) and 60 min (HL60) of HL exposure and during recovery at GL for 15 min (R15) and 60 min (R60) after 60 min of HL treatment. **A)** Concentration of glutathione (GSH) and oxidized glutathione (GSSG). **B)** Ratio of GSH to GSSG. **C)** Content of 12-oxo-phytodienoic acid conjugated to GSH (GS-OPDA). **D)** Concentration of ascorbate (AsA) and dehydroascorbate (DHA). **E)** Ratio of AsA to DHA. **F)** Concentration of H_2_O_2_. **G)** Concentration of TBARS. The concentrations were expressed as μmol **(A, D, F)**, nmol **(G)** and peak area **(C)** in g of leaf fresh weight (FW). Values represent the mean ± SD of 4 independent samples. Letters indicate significant differences between the treatments (ANOVA, Tukey-HSD, *P* < 0.05).

To assess the relationship between the gene expression and metabolite accumulation during the HL-exposure and subsequent R phase of the leaves at GL, we next analyzed the abundance of stress hormones, H_2_O_2_, and molecular antioxidants during the course of HL and R. Distinct patterns in metabolite concentrations were displayed throughout the experiment (Figs. 6 and 7). No significant changes were observed in the amounts of SA and ABA in HL, whereas both ABA and SA levels decreased during the R60 phase (Fig. 6, A and B). OPDA levels showed an initial increase at HL60, followed by a subsequent decrease during the R phase (Fig. 6C). In contrast, JA levels clearly increased during the HL treatment, and even more during the subsequent R phase (Fig. 6D).

The concentrations of molecular antioxidants were also measured to evaluate their role in response to HL and R treatments. GSH content remained unchanged during the HL treatment but showed a significant increase during the R phase (Fig. 7A). GSSG peaked at R15 and decreased at R60 (Fig. 7A). In addition, the amount of OPDA conjugated to GSH (GS-OPDA) increased during HL exposure and then decreased during R phase (Fig. 7C).

Amino acids with antioxidant properties were measured from leaves to assess their contribution to antioxidant capacity. Methionine levels increased with HL treatment and remained elevated during R phase (Supplementary Fig. S3). Homocysteine, tryptophan, and histidine levels spiked in HL60 and decreased during the R phase (Supplementary Fig. S3).

Reduced ascorbate (AsA) levels increased during R phase, and correspondingly, oxidized ascorbate (DHA) levels increased with HL treatment and decreased during R (Fig. 7D). H_2_O_2_ levels peaked at HL60 and decreased during R phase, whereas lipid peroxidation levels, as measured by thiobarbituric acid reactive substances (TBARS), increased during R phase (Fig. 7, F and G).

### Transcript accumulation of the genes encoding GSH metabolic enzymes during HL and subsequent recovery

**Figure 8.**
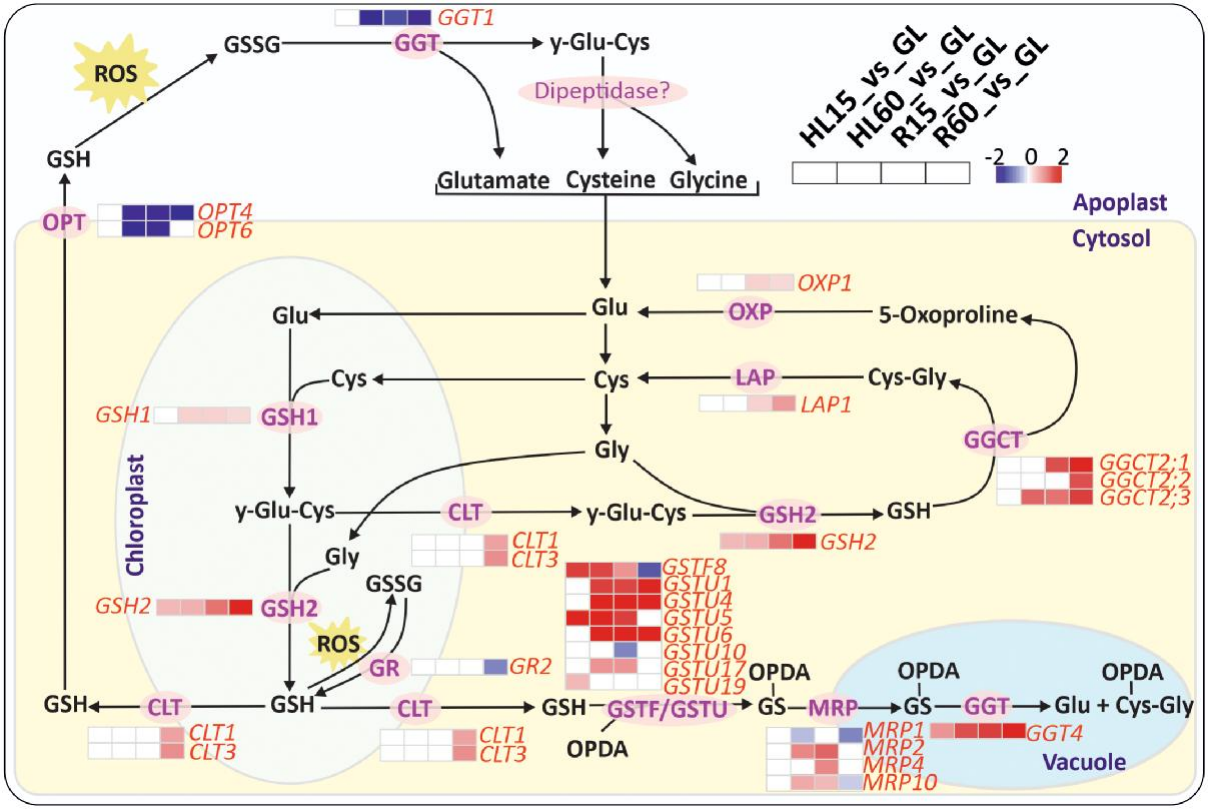
Differential expression of genes involved in glutathione (GSH) metabolism in leaves treated in high light (HL) and in recovery (R) at growth light (GL) in comparison to control GL leaves. HL treatment was performed by exposing plants to HL for 15 (HL15) and 60 min (HL60), while recovery was performed by transferring HL60 samples to GL for 15 (R15) and 60 min (R60) to recover. The illustration of GSH metabolism was modified from (Dorion et al. 2021). *GSTF/GSTU* genes involved in GSH conjugation of 12-oxo-phytodienoic acid (OPDA) were taken from (Skipsey et al. 2011). The red and blue color scale (log2-fold change) shows the degree of up- and downregulation of the genes, respectively. Only genes with statistically significant log2-fold changes in gene expression compared to GL (*P* < 0.05) are shown in the figure. Non-significant values were replaced by 0 in the heat map. The names of genes and corresponding proteins are listed in the text. For the accession numbers of the genes and log2-fold changes, see Supplementary Table S3.

Since the GSH levels were particularly elevated in R phase, we next analyzed the expression response of genes encoding proteins involved in GSH metabolism, after both HL and R phases. The genes related to GSH biosynthesis, cellular transport, and recycling were differentially expressed in HL and subsequent R phases (Fig. 8, Supplementary Table S3). The expression levels of *GLUTATHIONE SYNTATHASE 2 (GSH2)*, *GAMMA-GLUTAMYL CYCLOTRANSFERASE 2s (GGCT2), LEU AMINOPEPTIDASE 1 (LAP1),* and *ATP-DEPENDENT 5-OXOPROLINASE (OXP1)* were increased during R phase in comparison to GL (Fig. 8). *GSH2* encodes the GSH biosynthetic enzyme that is dually targeted to plastid and cytosol, and it catalyzes the addition of glycine (Gly) to y-glutamyl-cysteine (y-Glu-Cys) (Dorion et al. 2021), while *GGCT2s, LAP1*, and *OXP1* encode the cytosolic enzymes that degrade and recycle GSH (Dorion et al. 2021; Ito and Ohkama-Ohtsu 2023). GSH and y-Glu-Cys are exported from chloroplasts to the cytosol via CHLOROQUINE-RESISTANCE TRANSPORTER-LIKE TRANSPORTER (CLT) (Dorion et al. 2021). *CLT* genes were slightly upregulated in R60 (Fig. 8, Supplementary Table S3). Conversely, the expression of the *OLIGOPEPTIDE TRANSPORTER* and *GAMMA-GLUTAMYL TRANSPEPTIDASE 1* genes was significantly decreased in both HL and R phases compared to GL (Fig. 8). These genes encode proteins involved in the transport of GSH to the apoplast and the degradation of GSSG in the apoplast, respectively (Fig. 8) (Zhang et al. 2016; Wongkaew et al. 2018; Ito and Ohkama-Ohtsu 2023), suggesting that the transport of GSH to the apoplast was reduced by the HL and R treatments.

The expression of the genes related to OPDA conjugation with GSH and degradation of GS-OPDA in the vacuole was increased in both HL and R phases (Fig. 8, Supplementary Table S3). Eleven GLUTATHIONE S-TRANSFERASES (GSTs and GSTUs) have been shown to catalyze OPDA conjugation with GSH (Skipsey et al. 2011). Seven out of the 11 genes were upregulated in response to HL and R treatments (Fig. 8, Supplementary Table S3), suggesting that these genes are responsive to OPDA levels in cells (Fig. 6). In addition, *GGT4* gene was upregulated by HL and R treatments (Fig. 8). *GGT4* encodes the vacuolar isoform of GGT that initiates GS-OPDA degradation by catalyzing the cleavage of the y-Glu-Cys moiety from GS conjugates (Grzam et al. 2007; Dorion et al. 2021; Ito and Ohkama-Ohtsu 2023). We propose that GS-OPDA conjugate produced in the cytosol is transported to the vacuole via MULTIDRUG RESISTANCE-ASSOCIATED PROTEIN transporters for processing by GGT4 (Dorion et al. 2021; Ito and Ohkama-Ohtsu 2023). Thus, the DEGs associated with GSH metabolism suggest that GSH biosynthesis and recycling are critical for both HL and R processes.

### The *coi1* mutants show slightly faster PSII recovery after the HL treatment

Next, we investigated whether the lack of JA signaling has an effect on photosynthetic light reactions in plants exposed to HL and during subsequent recovery at GL. To this end, we used the *coi1* mutants lacking JA signaling (Fig. 4) and measured the photochemical efficiency of PSII (Fv/Fm) from Arabidopsis leaves. No significant differences in Fv/Fm were detected in HL-exposed plants, but during R phase at GL, Fv/Fm recovered slightly faster in the *coi1* mutants (Supplementary Fig. S4).

## Discussion

Relatively short exposures of Arabidopsis rosettes to HL, ranging from minutes to 1 h, followed by R periods of 15 or 60 min in GL, were applied here to mimic changes in light intensity under natural conditions (Fig. 1). Photosynthetic regulation mechanisms induced by changes in light intensity have been extensively investigated (Roach and Krieger-Liszkay 2012; Tikkanen et al. 2015; Yamori et al. 2015), as well as the expression of stress-responsive genes linked to genetic reprogramming in the plant cell during different durations of HL exposure (Dietz 2015; Gollan et al. 2015; Crisp et al. 2017; Huang et al. 2019; Zandalinas et al. 2020; Balcke et al. 2024; Eirich et al. 2024). However, much less is known about the production of signaling metabolites and gene expression profiles related to the R phase after exposure of plants to HL stress (Dietz 2015; Crisp et al. 2017; Gollan and Aro 2020). To gain specific information about the R phase, Arabidopsis leaves were subjected to global gene expression analysis and determination of the levels of ROS, stress hormones, and antioxidants using the leaves that were (i) maintained at GL, (ii) then transferred to HL for short periods of time, and (iii) finally transferred back to GL for 15 or 60 min for R.

### Short-term HL stress induces hydrogen peroxide, ABA, and oxylipin signals with related gene expression changes

To gain insight into the triggers underlying gene expression changes during HL stress and R, we first traced the components responsible for initiating the HL-responsive signaling cascades in Arabidopsis leaves. Compared to GL-maintained leaves, HL gradually induced H_2_O_2_ accumulation in leaves (Fig. 7F), which is generally reported to be counteracted by the production of antioxidants (Gechev et al. 2002; Hernández et al. 2004; König et al. 2018; Hieno et al. 2019). Although no substantial increase in GSH or ASA content was observed after HL exposure of Arabidopsis, the proportion of oxidized forms of glutathione (GSSG, HL15) and ascorbate (DHA, HL60) increased in leaves (Fig. 7, B and E), strongly suggesting that GSH and ASA were used for protection against oxidative stress, in agreement with previous reports (Yoshimura et al. 2000; König et al. 2018; Alvarez-Fernandez et al. 2021). Similarly, an increase in antioxidant amino acids also plausibly contributed to ROS scavenging in HL (Supplementary Fig. S3) (Khorobrykh et al. 2020). On the contrary, there were no signs of lipid peroxidation as detected by TBARS-reactive substances (Fig. 7G), thus eliminating the risk of severe oxidative stress in the cells. Instead, it is conceivable that the gradual accumulation of H_2_O_2_ allows its use as a signal to regulate gene expression and to induce protective processes to limit further increases in ROS levels.

Although H_2_O_2_ accumulation was only detected in HL60 leaves (Fig. 7F), the upregulation of several H_2_O_2_-responsive genes, such as HSPs, was evident after both 15 and 60 min of HL exposure (Fig. 5B and Supplementary Fig. S1B). These data suggest that H_2_O_2_ is one of the signals produced during leaf exposure to HL, which has been shown to be produced in PSI (Fitzpatrick et al. 2022; Tiwari et al. 2024). Singlet oxygen is also produced in HL (Triantaphylidès et al. 2008; Dmitrieva et al. 2020), while it is very efficiently scavenged by carotenoids and tocopherol (Ramel et al. 2012; Dmitrieva et al. 2020), which is likely reflected in the strong differences in ^1^O_2_-responsive gene expression between 15 and 60 min duration of the HL treatments (Supplementary Fig. S1A). In this work, OPDA and JA biosynthesis genes were already moderately to highly upregulated in HL (Fig. 3), and this increased expression was reflected in the accumulation of oxylipins in leaves (Fig. 6). A portion of ABA-responsive genes was also upregulated by HL treatment (Supplementary Fig. S2A).

Taken together, we conclude that H_2_O_2_, ABA, and oxylipins are among the compounds produced by short-term HL treatment and involved in the initiation of the respective signaling cascades. This is consistent with previous reports highlighting the role of H_2_O_2_-, ABA- and JA-responsive genes in HL stress (Dietz 2015; Balfagón et al. 2019; Hieno et al. 2019; Huang et al. 2019; Zandalinas et al. 2020; Alvarez-Fernandez et al. 2021).

### JA signaling gains importance in recovery from short-term HL stress

**Figure 9.**
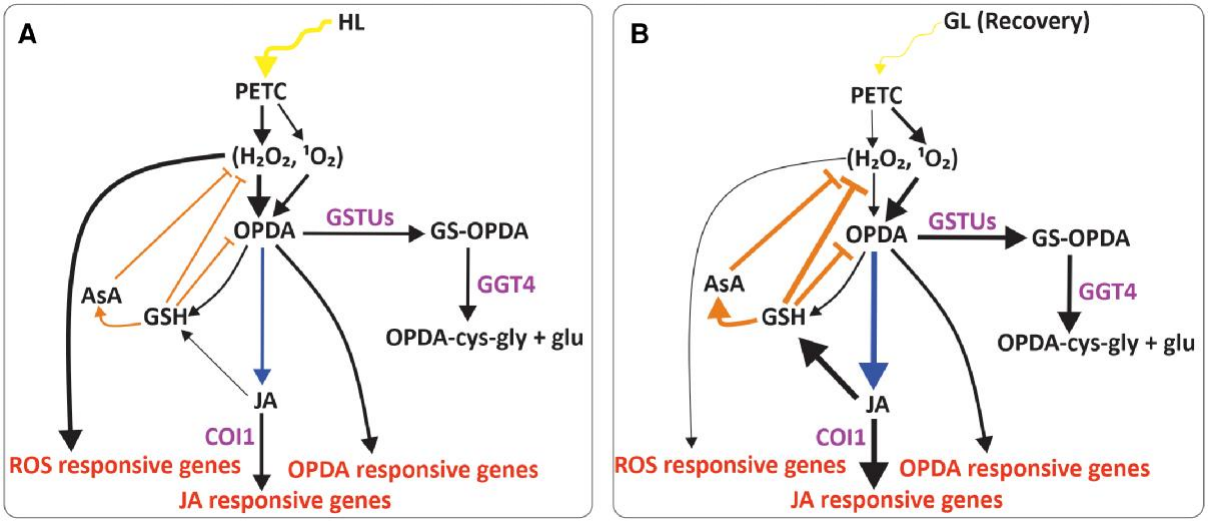
A model of signaling pathways induced by high light (HL) and during subsequent recovery at growth light (GL). **A)** HL triggers the accumulation of reactive oxygen species (H_2_O_2_ and ^1^O_2_), which leads to activation of ROS-responsive genes. Limited levels of reduced glutathione (GSH) and reduced ascorbic acid (ASA) allow ROS signaling to occur. In addition, ROS activate pathways that lead to the synthesis of oxylipins. This results in the accumulation of 12-oxo-phytodienoic acid (OPDA), which is subsequently converted to jasmonic acid (JA). Both OPDA and JA stimulate the expression of oxylipin-responsive genes. **B)** Upon return to GL for recovery from HL stress, accumulation of H_2_O_2_ decreases, while production of ^1^O_2_ continues for some time, resulting in continued synthesis of JA via OPDA. OPDA does not accumulate, because, in addition to its conversion to JA, it is scavenged by GSH via GLUTATHIONE S-TRANSFERASE (GSTU) activity and the GS-OPDA conjugates are processed in vacuoles by GAMMA-GLUTAMYL TRANSPEPTIDASE 4 (GGT4). OPDA and JA stimulate the synthesis of GSH and ASA, which scavenge ROS and restore the redox state of the cells, thereby promoting recovery. The thickness of the arrow indicates the intensity of the pathway. CORONATINE INSENSITIVE 1 (COI1).

The enzymatic synthesis of oxylipins begins with the oxidation of polyunsaturated fatty acids, a process that is enhanced by oxidative stress (Montillet et al. 2004; Knieper et al. 2023). Accordingly, HL has been shown to upregulate oxylipin biosynthesis and increase JA production (Balfagón et al. 2019; Huang et al. 2019), possibly via ROS accumulation (Fig. 9). The initiation of oxylipin synthesis in chloroplasts has been linked to the production of ^1^O_2_ and H_2_O_2_ in photosynthesis (Fischer et al. 2013; Hieno et al. 2019; Lv et al. 2019; Gollan and Aro 2020). In this work, the genes involved in oxylipin biosynthesis were slightly upregulated in HL, while the expression of the genes was further enhanced during the R after HL treatment (Fig. 3A) (Gollan and Aro 2020). This upregulation of the biosynthetic genes also leads to a high accumulation of JA at R60 (Fig. 6). We propose that oxylipins function as a molecular memory that continues to reprogram gene expression after HL stress and guides the recovery process at R period (Fig. 9). This conclusion was further strengthened by using the JA receptor *coi1* mutants, which showed a lack of expression of R-sustained genes (Fig. 4), thereby confirming the involvement of JA signaling in R phase.

In nature, plants are continuously exposed to fluctuating light with recurrent changes in low, moderate, and high irradiance. Photosynthetic regulatory mechanisms respond to these changes in light intensity, while JA may also be involved in protecting against harmful changes caused by light fluctuations. This means that during the day, JA signaling remains active for a longer period of time and is used to prepare for an upcoming increase in light intensity. Such a molecular memory based on the microRNA pathway has been observed to function during heat stress in Arabidopsis (Stief et al. 2014). Here, we observed that the duration of the HL pretreatment that induced upregulation of a set of R-sustained genes was remarkably short (Fig. 5A). Such a rapid release of signal in HL, triggering downstream targets upon subsequent transfer of plants to lower light intensity, has been reported previously (Dietz 2015). This rapid response suggests that even very brief exposure to HL, such as canopy movements, may be long enough to induce JA signaling to control metabolism once typical canopy conditions have returned.

The primary signal generated by HL that leads to JA accumulation, both in HL and especially when plants are returned to GL conditions, has remained elusive. Both H_2_O_2_ and ^1^O_2_ trigger oxylipin biosynthesis (Fischer et al. 2013; Hieno et al. 2019; Gollan and Aro 2020), but here we show a decrease in H_2_O_2_ levels back to the control levels and a decrease in H_2_O_2_ signaling already within 15 min in R conditions (Fig. 7F and Supplementary Fig. S1). The ROS produced at the beginning of R could be ^1^O_2_, since the expression of ^1^O_2_-induced genes peaked at R15 (Supplementary Fig. S1A). This is further supported by the observation that the accumulation of TBARS (Fig. 7G), indicative of lipid peroxidation, continued during R phase. Thus, it is possible that OPDA was still produced in chloroplasts during R phase, but the total amount decreased due to its high conversion to JA (Fig. 6). Photodamaged PSII complexes could be the source of ^1^O_2_ (Krieger-Liszkay 2005), since Fv/Fm decreased by about 20% during HL treatment and damaged PSII complexes were only gradually repaired at GL (Supplementary Fig. S4) (Rintamäki et al. 1996). Surprisingly, we observed that at GL after HL stress, Fv/Fm in *coi1* mutants was restored slightly faster than in WT (Supplementary Fig. S4). The faster recovery in *coi1* mutants may be caused by the JA-induced growth retardation and induction of defense processes (Sasaki-Sekimoto et al. 2005; Bali et al. 2023) that occurs in WT but is absent in *coi1* mutants. Immediately after the transfer of the plants from HL back to GL, *coi1* mutants may have more resources to be used to repair the photoinhibited PSII centers because of the insufficient induction of the defense processes in mutants lacking the JA receptor.

### Glutathione and ascorbate are key antioxidants to ensure the recovery from HL stress

When Arabidopsis plants were transferred from HL stress to R conditions, a drastic increase in GSH and AsA levels was observed in leaves, and the GSH/GSSG and AsA/DHA ratios were rapidly restored to control levels (Fig. 7), suggesting a critical role for these antioxidants in R processes. JA has been shown to activate the genes involved in GSH and AsA biosynthesis (Sasaki-Sekimoto et al. 2005; Han et al. 2013), suggesting a synergy between JA signaling and the levels of these antioxidants during recovery (Han et al. 2013). JA also induces the expression of genes involved in sulfur assimilation into Cys, an upstream step in GSH synthesis (Jost et al. 2005; Sasaki-Sekimoto et al. 2005). In addition, OPDA induces GSH production by facilitating the binding of CYP20-3 to the cysteine synthase complex (Park et al. 2013; Liu et al. 2020). The increase in the expression of genes encoding enzymes involved in GSH synthesis (*GSH2*) and recycling (*LAP1*, *OXP1*, *GGCT2*) corresponded with GSH accumulation during the R phase (Fig. 8). On the other hand, GSH neutralizes the RES activity of OPDA through glutathionylation of unsaturated double bonds, after which GS-OPDA is targeted for degradation in the vacuole (Dorion et al. 2021; Ito and Ohkama-Ohtsu 2023). Production of GS-OPDA conjugates was highest in HL60, where OPDA also highly accumulated (Figs. 6, C and 7C), suggesting that GS-OPDA conjugation protects cells from the toxic effects of OPDA (Knieper et al. 2023). Furthermore, the concomitant decrease in OPDA and GS-OPDA conjugate during R suggests that in addition to the conversion of OPDA to JA, it is processed in the vacuole by GGT4 (Fig. 8).

AsA is another important antioxidant in plant metabolism (Foyer and Noctor 2011; Noctor et al. 2024). It is linked to GSH metabolism via the ascorbate-glutathione cycle, which scavenges H_2_O_2_ (Foyer and Noctor 2011). Like GSH, AsA levels did not increase during the HL exposure, as previously reported (Balcke et al. 2024), but the content in leaves almost doubled under R conditions at GL (Fig. 7D). It is conceivable that plants do not invest in the synthesis of AsA and GSH during the HL stress and instead prefer the production of ROS for signaling purposes. Conversely, when the HL stress is terminated by shifting plants to R conditions, the increase in AsA and GSH synthesis is crucial for the detoxification of ROS (Fig. 9).

## Conclusions

As demonstrated in Fig. 9, we propose insights into the signaling mechanisms used during the R phase after short-term HL stress. H_2_O_2_ and ^1^O_2_ accumulate in HL, leading to the synthesis of OPDA, which is used for JA synthesis, especially during the R phase. JA acts as a molecular memory in the R phase by inducing the accumulation of GSH and AsA, which scavenge the ROS and restore the redox state of the cells, thereby promoting recovery from the stress. Remarkably, even a short exposure to HL stress, as little as 2 min, is sufficient to initiate some of the signaling cascades typical of the R phase.

## Materials and methods

### Plant material, growth conditions, and light treatments

WT plants of Arabidopsis were used to study signaling initiated by HL treatment and in subsequent R phase at GL. WT and *coi1* mutants were used for the study of JA signaling during R from HL stress. *coi1-1* and *coi1-2* mutant lines at Col-0 background were obtained from The Nottingham Arabidopsis Stock Centre (NASC, Nottingham, UK, N68754 and N68755).

Plants were grown under 8 h of light (100 μmol photons m^−2^ s^−1^) and 16 h darkness for 6 wk in a phytotron growth chamber. Temperature, CO_2_ concentration, and humidity were 23 °C, 400 ppm and 60%, respectively. OSRAM PowerStar HQIT 400/D metal halide lamps (Osram, Munich, Germany) were used for illumination during growth. Leaf number 7 from Arabidopsis rosettes was used for analysis of metabolites and gene expression.

For transcriptomics and metabolite quantification, WT plants were exposed to 1000 μmol photons m^−2^ s^−1^ for 15 min (HL15) or 60 min (HL60), while subsequent recovery from HL60 involved incubation under GL (100 *μ*mol photons m^−2^ s^−1^) for 15 min (R15) or 60 min (R60) (Fig. 1A).

COI1-dependence of the expression of JA marker genes in WT, *coi1-1* and *coi1-2* mutant lines was assessed by RT-qPCR analysis. GL samples were harvested before the HL treatment of 60 min, and the R60 samples were harvested from plants transferred to GL for 60 min after the HL treatment for RT-qPCR analyses (Fig. 1B).

The duration of the HL pretreatment required to enhance the expression of R-sustained marker genes was determined by RT-qPCR analysis. Leaves of WT plants were harvested from GL and after the HL treatment of 2 (HL2), 5 (HL15) and 60 (HL60) min. Following the HL treatment, plants were transferred to GL for recovery for 15 min (R15) and 60 min (R60) (Fig. 1C).

### Measurement of metabolites and glutathione-OPDA conjugate with ultra-performance liquid chromatography

Plant leaves were frozen with liquid nitrogen and ground with mortar to a fine powder. 1 ml of methanol was added to the leaf powder. The samples were subjected to homogenization for 30 s. The suspension was centrifuged at 4 °C for 5 min at 14,000*×g*. 800 *μ*l of supernatant was transferred to an Eppendorf tube for ultra-performance liquid chromatography (UPLC) analysis. UPLC analyses were performed by the Turku Metabolomics Centre (Turku Bioscience Centre, Turku, Finland). The standards for hormones and amino acids were obtained from Sigma-Aldrich (Sigma-Aldrich, St. Louis, MO, USA). The concentrations were expressed as μg in g of leaf fresh weight (FW). The standard for glutathione-OPDA (GS-OPDA) conjugate was obtained from the reaction between GSH and OPDA (Dixon and Edwards 2009) and the concentration was expressed as peak area per g of FW.

### H_2_O_2_ and TBARS measurements

H_2_O_2_ measurement was carried out according to the method described previously (Bela et al. 2018). Leaves were frozen with liquid nitrogen and ground with mortar into a fine powder. 500 *μ*l of 0.1% trichloroacetic acid (TCA) was added to the leaf powder. The suspension was centrifuged at 7000*×g* for 20 min at 4 °C. 250 *μ*l of supernatant was transferred to an Eppendorf tube and 500 *μ*l of 50 mm potassium phosphate buffer (pH 7.0) and 0.5 ml of 1 m potassium iodide were added. Following incubation of 10 min at 25 °C, the absorbance of the samples was measured at 390 nm. Concentration of H_2_O_2_ in the sample was calculated by using a standard curve with known concentrations of H_2_O_2_ and results expressed as μmol H_2_O_2_ in g of FW.

TBARS were measured as described previously (Lima-Melo et al. 2019) to assess the level of lipid peroxidation as an indicator of oxidative stress. The concentrations were expressed as nmol TBARS in g of FW.

### Glutathione measurement

Glutathione measurements were carried out according to the method described previously (Bela et al. 2018). Plant samples were frozen with liquid nitrogen and ground with a mortar to a fine powder. 1.2 ml of 5% TCA was added to the powder and the suspension was centrifuged at 13,000*×g* for 20 min. For the glutathione assay, 100 *μ*l of the supernatant was added to either 100 *μ*l of water (for the total glutathione assay) or 100 *μ*l of 10% 2-vinylpyridine (for the GSSG assay, to mask GSH). 770 *μ*l of 100 mm phosphate buffer (pH 7.5) containing 10 *μ*l each of 1 mm 5,5′-dithio-bis-(2-nitrobenzoic acid), 1 mm NADPH, and 1 unit of GR (Sigma-Aldrich, St. Louis, MO, USA), was added to the mixture and thoroughly mixed. The absorbance of the reaction mixture was measured at 412 nm. The GSH content was calculated by subtracting GSSG concentration from the concentration of total glutathione. The concentration of glutathione in the sample was calculated by a standard curve that was prepared utilizing solutions of glutathione with known concentrations. The concentrations were expressed as μmol in g of FW.

### Ascorbate measurement

Ascorbate levels were measured as described previously (Zhang et al. 2009). Leaf samples were frozen with liquid nitrogen and ground with mortar to a fine powder to which 1.2 ml of 5% TCA was added. The suspension was centrifuged at 13,000*×g* for 20 min. For the determination of total ascorbate, 100 *μ*l of the supernatant were mixed with 100 *μ*l of 10 mm dithiothreitol (DTT). After 10 min incubation, 100 *μ*l of 0.5% *N*-ethylmaleimide (NEM) was added into the mixture and incubated for 15 min. The suspension was neutralized by adding 50 *μ*l of 1 m sodium hydroxide followed by an addition of 100 *μ*l of 0.1 m potassium ferricyanide (K_3_Fe(CN)_6_) in 50 mm phosphate buffer (pH 7.0) and 1 ml of the 0.1 m ferric chloride (FeCl_3_) solution. For reduced ascorbate (AsA), 200 *μ*l of water was added instead of DTT and NEM. The reaction solution was incubated at 37 °C for 30 min and the absorbance of the solution was measured at 735 nm. The concentration of oxidized ascorbate (DHA) was calculated by subtracting the concentration of AsA from the concentration of total ascorbate. The concentration of ascorbate in the sample was calculated by a standard curve with known concentrations of ascorbate. The concentrations were expressed as μmol in g of FW.

### RNA isolation and analysis

The leaf samples were frozen in liquid nitrogen and ground in a mortar, followed by RNA extraction using the innuPREP Plant RNA Kit (Analytik Jena, Jena, Germany) according to the kit instructions.

For RNAseq, the RNA extracts were sent to BGI Europe Genomic Center (Copenhagen, Denmark) for sequencing. Transcript reads of 3 biological replicates were quantified with Salmon (v0.12) software using cDNA sequences from The Arabidopsis Information Resource database (TAIR) for reference. Statistical analysis of differential gene expression was carried out with Bioconductor DESeq2 package (Love et al. 2014). Differentially expressed genes (DEGs) were annotated using the TAIR 10.49.gtf. Genes with read counts lower than 10 were eliminated before the differential expression analysis was performed. DEGs were identified by Wald tests, and the obtained *P*-values were adjusted for multiple testing using the Benjamini-Hochberg correction to control the false discovery rate. DEGs with adjusted *P*-value (padj) < 0.05 were selected to create the Venn diagrams and the heatmaps. Gene enrichment analysis was performed with http://geneontology.org/software (accessed on June 15, 2023). Genes with expression of −1 ≥ log2(FC) ≥ 1 were selected for gene enrichment and Venn diagram analysis.

For RT-qPCR, iScript cDNA Synthesis Kit (Biorad, Hercules, CA, USA) was used to synthesize cDNA from RNA extract. The cDNA solution was diluted fivefold with water before use. RT-qPCR measurements were carried out with a Biorad iq5 real time PCR machine (Biorad, Hercules, CA, USA). RT-qPCR samples contained 10 *µ*l of SYBR Green PCR Master Mix (Biorad, Hercules, CA, USA), 0.5 *µ*l of cDNA, 1 *µ*l of forward primer (10 *µ*M), 1 *µ*l of reverse primer (10 *µ*M) and 3 *µ*l of water. Primers (Supplementary Table S4) for the reference gene UBIQUITIN CONJUGATING ENZYME 9 (UBC9) and marker genes were designed with QuantPrime (accessed on February 10, 2023) and obtained from Sigma Aldrich (St. Louis, MO, USA).

### Photosynthetic efficiency of PSII

The photosynthetic efficiency of PSII (Fv/Fm) in WT and the *coi1* mutant was measured from the seventh leaf of plants with Dual-PAM-100 (Heinz Walz GmbH, Effeltrich, Germany) according to (Kılıç et al. 2023). The plants were dark acclimated for 20 min before measurements.

### Statistical analyses

Statistical significance of metabolite and RT-PCR data was tested by using a 1-way ANOVA with the post hoc Tukey HSD Calculator (https://astatsa.com, accessed on April 30, 2024). The number of biological replicates is indicated in the figure legends.

### Accession numbers

Sequence data used in this article can be obtained from TAIR database based on the accession numbers indicated in the Figures and Supplementary Data.

## Supplementary Material

kiaf143_Supplementary_Data

## Data Availability

The data supporting the findings of this study are available in the article and its Supplementary materials. The transcriptome data have been deposited to the NCBI GEO repository under GSE277977.

## Supplementary data

The following materials are available in the online version of this article.

**Supplementary Figure S1.** Differential expression of singlet oxygen (^1^O_2_) and hydrogen peroxide (H_2_O_2_) -responsive genes in HL and R at GL in comparison to GL.

**Supplementary Figure S2.** Differential expression of ABA and salicylic acid (SA)-responsive genes in HL and R at GL in comparison to GL.

**Supplementary Figure S3.** Content of antioxidant amino acids in leaves exposed to HL and subsequently moved to R at GL.

**Supplementary Figure S4.** Photochemical efficiency of photosystem II (Fv/Fm) in leaves of WT and *coi1* mutants (*coi1-1* and *coi1-2*) exposed to different durations of HL and under subsequent R at GL.

**Supplementary Table S1.** Gene enrichment analysis of biological processes upregulated in HL and R at GL in comparison to GL control conditions.

**Supplementary Table S2.** Average transcript counts of marker genes in RNAseq analyses selected for the RT-qPCR assay presented in Figs. 4 and 5.

**Supplementary Table S3.** Differential expression of genes encoding glutathione metabolic enzymes in leaves treated in HL and R condition at GL in comparison to GL.

**Supplementary Table S4.** Marker genes and the primers used in the RT-qPCR assay.

## Dive Curated Terms

The following phenotypic, genotypic, and functional terms are of significance to the work described in this paper:

UBC9 Gramene: AT5G41700
UBC9 Araport: AT5G41700

VSP2 Gramene: AT5G24770
VSP2 Araport: AT5G24770

GGT4 Gramene: AT4G29210
GGT4 Araport: AT4G29210

AOC2 Gramene: AT3G25770
AOC2 Araport: AT3G25770

GSH2 Gramene: AT5G27380
GSH2 Araport: AT5G27380

OXP1 Gramene: AT5G37830
OXP1 Araport: AT5G37830

JAR1 Gramene: AT2G46370
JAR1 Araport: AT2G46370

glutathione CHEBI: CHEBI:16856

COI1 Gramene: AT2G39940
COI1 Araport: AT2G39940

JAZ8 Gramene: AT1G30135
JAZ8 Araport: AT1G30135

JAZ10 Gramene: AT5G13220
JAZ10 Araport: AT5G13220
