# Supplementary material for: Jasmonic acid signaling and glutathione coordinate plant recovery from high light stress
Source: Plant Physiol. 2025 Apr 10;197(4):kiaf143. doi: 10.1093/plphys/kiaf143 (PMC12038154; doi:10.1093/plphys/kiaf143)
Supplement: kiaf143_Supplementary_Data [file kiaf143_supplementary_data.pdf]

SUPPLEMENTARY DATA

Jasmonic acid signaling and glutathione coordinate plant recovery from high light stress

Mehmet Kılıç, Peter J. Gollan, Eva-Mari Aro, and Eevi Rintamäki

Supplementary Figures:

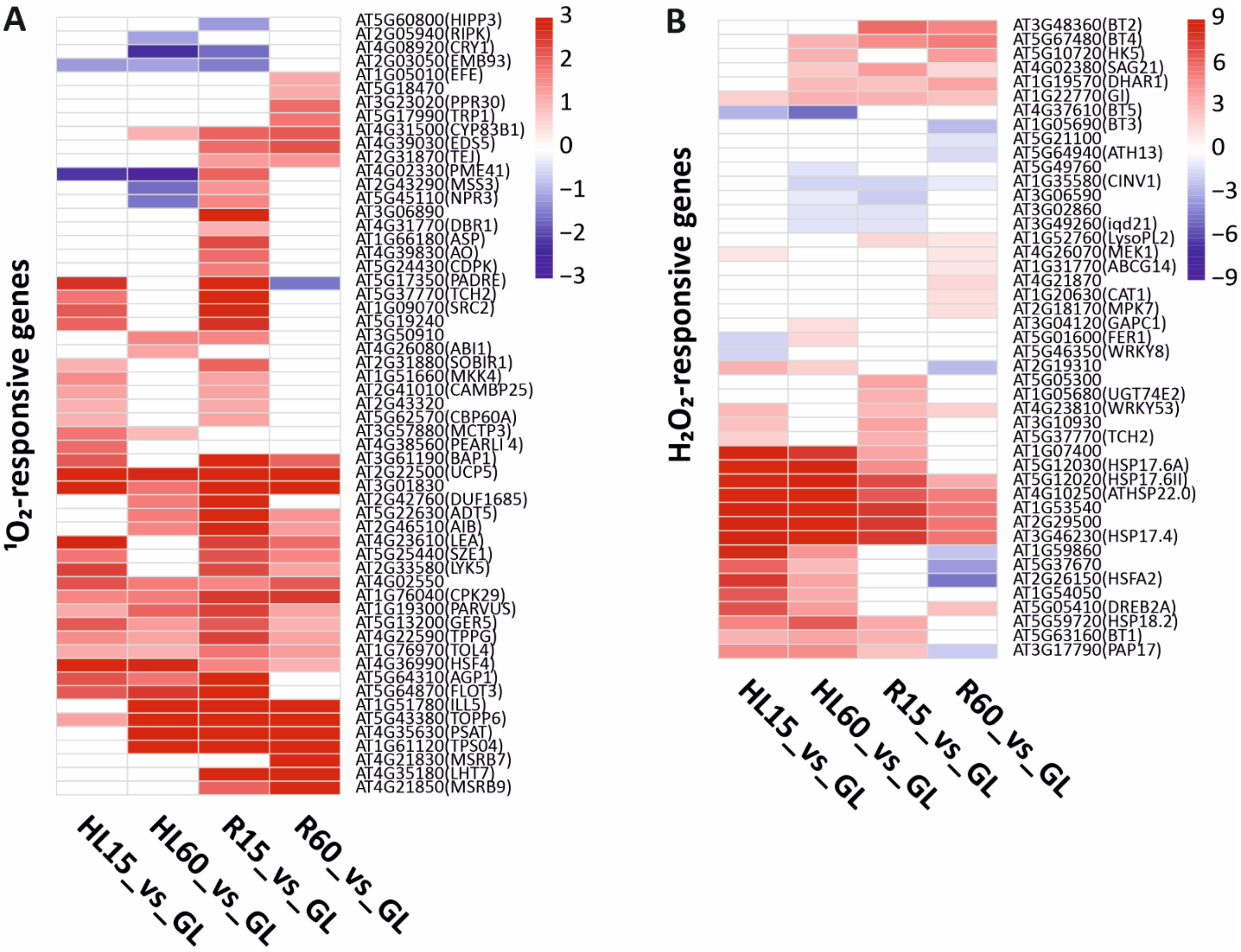

Supplementary Figure S1. Differential expression of singlet oxygen ($^1O_2$) and hydrogen peroxide (H_2O_2) - responsive genes in high light (HL) and recovery (R) at growth light (GL) in comparison to GL. HL treatment was performed by exposing plants to HL for 15 (HL15) and 60 min (HL60), while recovery was performed by transferring HL60 samples to GL to recover for 15 (R15) and 60 (R60) min. **A)** Differential expression of genes responsive to $^1O_2$. The genes included in the figure are reported to be upregulated in response to $^1O_2$ in (Op Den Camp et al., 2003) with the addition of genes included in the following GO terms: singlet

oxygen-mediated programmed cell death (GO:0010343), response to singlet oxygen (GO:0000304), cellular response to singlet oxygen (GO:0071452). **B)** Differential expression of genes responsive to H_2O_2. The genes included in the figure are reported to be upregulated in response to H_2O_2 treatment in (Gollan and Aro, 2020). Red and blue color scale (log2-fold change) shows the degree of upregulation and downregulation of the genes, respectively. The scale of $^1O_2$ responsive genes is from -3 to 3, while the scale of H_2O_2 genes is from -9 to 9. Only the statistically significant log2-fold changes ($p < 0.05$) of gene expression in comparison to GL are shown in the figure. Non-significant values were replaced by 0 in the heat map.

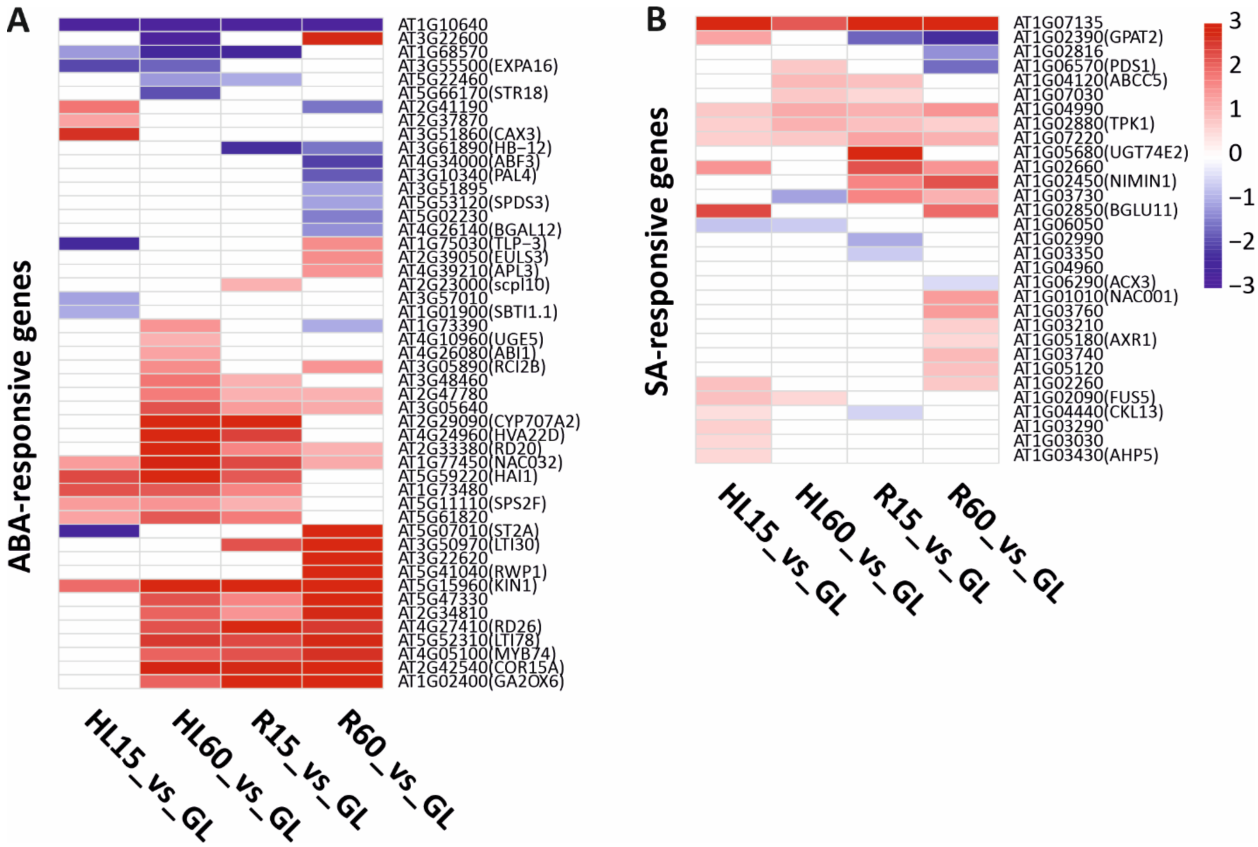

Supplementary Figure S2. Differential expression of abscisic acid (ABA) and salicylic acid (SA) -responsive genes in high light (HL) and recovery (R) at growth light (GL) in comparison to GL. HL treatment was performed by exposing plants to HL for 15 (HL15) and 60 min (HL60), while recovery was performed by transferring HL60 samples for 60 min to GL to recover for 15 (R15) and 60 (R60) min. **A)** Differential expression of genes responsive to ABA. The genes included in the figure are reported to be upregulated in response to ABA treatment in (Xin et al., 2005). **B)** Differential expression of genes responsive to SA. The genes included in the figure are reported to be upregulated in response to SA (Zhang et al., 2020). Red and blue color scale (log2-fold change) shows the degree of upregulation and downregulation of the genes, respectively. Only the statistically significant log2-fold changes ($p < 0.05$) of gene expression in comparison to GL are shown in the figure. Non-significant values were replaced by 0 in the heat map.

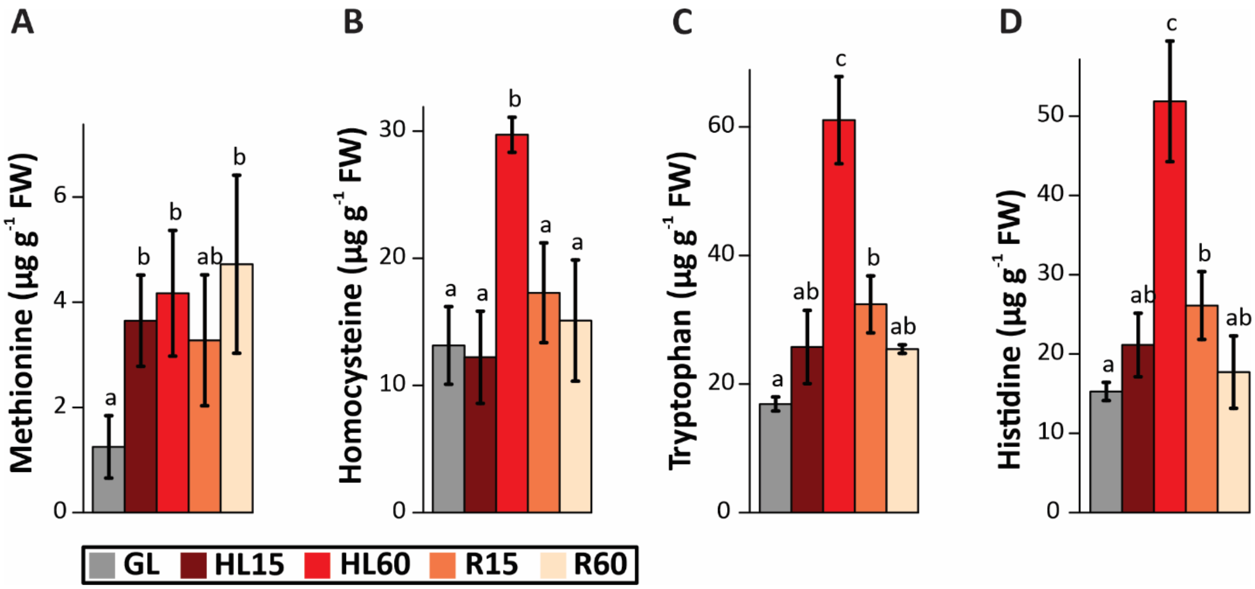

Supplementary Figure S3. Content of antioxidant amino acids in leaves exposed to high light (HL) and subsequently moved to recover (R) at growth light (GL). **A)** Methionine. **B)** Homocysteine. **C)** Tryptophan. **D)** Histidine. Measurements were done with leaves taken from plants before the HL treatment (GL), after 15 min (HL15) and 60 min (HL60) of HL exposure, and during the recovery at GL for 15 min (R15) and 60 min (R60) after 60 min HL treatment. The values represent the mean ± SD of four independent samples. The concentrations were expressed as µg in g of leaf fresh weight (FW). Letters indicate significant differences between the treatments (ANOVA, Tukey-HSD, $p < 0.05$).

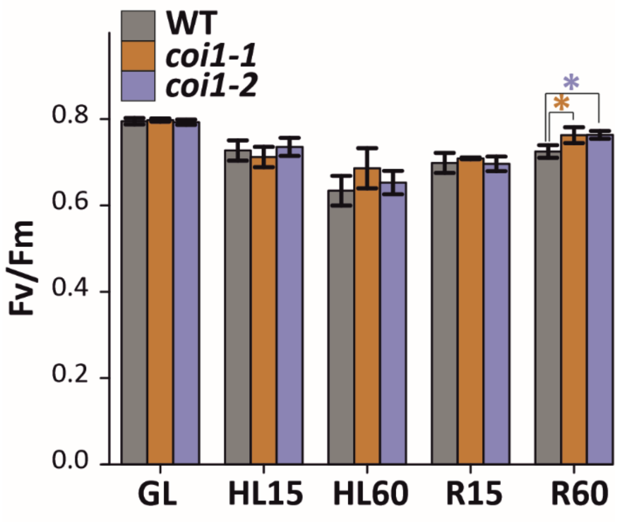

Supplementary Figure S4. Photochemical efficiency of photosystem II (Fv/Fm) in leaves of wild type (WT) and *coi1* mutants (*coi1-1* and *coi1-2*) exposed to different durations of high light (HL) and under subsequent recovery (R) at growth light (GL). The Fv/Fm values were measured from plants before the HL treatment (GL), after 15 (HL15) and 60 (HL60) min of HL, as well as during recovery under GL conditions for 15 (R15) and 60 (R60) min after 60 min HL treatment. Values represent the mean ± SD of four independent samples. Asterisk (*) indicates significant difference between WT and *coi1* mutants (ANOVA, Tukey-HSD, $p < 0.05$).

Supplementary Tables:

Supplementary Table S1. Gene enrichment analysis of biological processes upregulated in high light (HL) and recovery (R) at growth light (GL) in comparison to GL control conditions.

		Fold Enrichment			
**GO biological process**	**GO term ID**	**HL15_vs_GL**	**HL60_vs_GL**	**R15_vs_GL**	**R60_vs_GL**
Response to hydrogen peroxide	GO:0042542	**7.6**	**5.2**	**3.7**	**3.1**
Response to reactive oxygen species	GO:0000302	**5.7**	**4.2**	**3.4**	**2.9**
Response to oxidative stress	GO:0006979	**4.1**	**2.8**	**3.5**	**2.5**
Glutathione metabolic process	GO:0006749	1.9	**3.3**	**2.8**	**3.0**
Regulation of jasmonic acid mediated signaling pathway	GO:2000022	**6.7**	**5.8**	**6.5**	**5.3**
Jasmonic acid mediated signaling pathway	GO:0009867	**4.9**	**6.4**	**6.4**	**4.6**
Cellular response to jasmonic acid stimulus	GO:0071395	**4.4**	**6.5**	**6.6**	**4.8**
Response to jasmonic acid	GO:0009753	**3.6**	**5.2**	**5.5**	**4.2**
Jasmonic acid biosynthetic process	GO:0009695	4.9	**7.6**	**5.4**	**5.8**
Jasmonic acid metabolic process	GO:0009694	2.8	**6.4**	**4.4**	**5.1**
Abscisic acid-activated signaling pathway	GO:0009738	**3.4**	1.8	**2.5**	1.5
Cellular response to abscisic acid stimulus	GO:0071215	**3.1**	1.6	**2.3**	1.4
Response to abscisic acid	GO:0009737	**3.6**	**2.6**	**2.7**	**2.0**
Regulation of abscisic acid-activated signaling pathway	GO:0009787	**3.0**	1.7	1.9	1.5
Regulation of salicylic acid metabolic process	GO:0010337	**7.8**	< 0.01	**3.7**	0.6
Salicylic acid mediated signaling pathway	GO:0009863	**6.0**	**2.8**	**5.8**	**2.9**
Cellular response to salicylic acid stimulus	GO:0071446	**5.9**	2.1	**5.7**	**2.7**
Response to salicylic acid	GO:0009751	**6.5**	**2.1**	**4.8**	**2.9**

Columns: 15 min HL (HL15), 60 min HL (HL60), 15 min R under GL (R15) and 60 min R under GL (R60) after 60 min HL treatment. Differentially expressed genes (DEGs) with log2-fold change (FC) value higher than 1 (log2 FC >1, p value < 0.05) were selected for gene enrichment analysis. Statistically significant enrichments (p < 0.05) are bolded.

Supplementary Table S2. Average transcript counts of marker genes in RNAseq analyses selected for the reverse transcription quantitative PCR (RT-qPCR) assay presented in Figures 4 and 5.

Gene ID	Name	GL	HL15	HL60	R15	R60
AT5G24770	VSP2	1065.4	2571.0	16746.9	11248.3	59152.7
AT3G25770	AOC2	2982.8	6536.8	18875.4	14594.6	31774.2
AT5G42650	AOS	9853.9	14076.2	31651.4	37196.3	58265.2
AT1G30135	JAZ8	4.5	21.2	31.0	532.6	52.6
AT1G43160	Rap2.6	4.5	1.0	313.0	76.2	320.6
AT5G13220	JAZ10	30.8	55.4	614.6	1358.2	674.4
AT2G26150	HSFA2	131.4	27578.3	1667.2	63.1	5.2
AT3G46230	HSP17.4A	26.1	9659.2	17214.8	5670.6	1127.7
AT1G53540	HSP17.6C	15.9	7391.8	13829.4	3415.2	671.9
AT1G52560	HSP 26.5	1.4	323.1	53.7	4.8	0.0
AT5G52640	HSP 90.1	787.6	75436.9	16674.6	1048.5	114.3

Columns present the transcript counts in RNAseq analysis of leaves taken from plants before the HL treatment (GL), after 15 min (HL15) and 60 min (HL60) of HL exposure, and during the recovery at GL for 15 min (R15) and 60 min (R60) after 60 min HL treatment.

Supplementary Table S3. Differential expression of genes encoding glutathione (GSH) metabolic enzymes in leaves treated in high light (HL) and recovery (R) condition at growth light (GL) in comparison to GL.

**Gene ID**	**Gene name**	**HL15_vs_GL**	**HL60_vs_GL**	**R15_vs_GL**	**R60_vs_GL**
AT4G39640	GGT1	-0.22	**-1.71**	**-1.30**	**-2.15**
AT4G29210	GGT4	**0.86**	**1.53**	**1.66**	**2.31**
AT4G23100	GSH1	0.11	**0.28**	**0.30**	**0.23**
AT5G27380	GSH2	**0.47**	**0.59**	**1.17**	**2.31**
AT2G24200	LAP1	0.13	0.19	**0.30**	**0.74**
AT5G37830	OXP1	0.25	0.15	**0.27**	**0.25**
AT5G19380	CLT1	0.09	0.37	0.52	**0.69**
AT5G12170	CLT3	0.05	0.22	0.13	**0.88**
AT5G26220	GGCT2;1	0.64	1.10	**1.51**	**3.27**
AT4G31290	GGCT2;2	-0.53	0.72	0.57	**1.52**
AT1G44790	GGCT2;3	0.38	**1.31**	**1.19**	**1.65**
AT3G54660	GR2	-0.09	-0.27	-0.11	**-0.86**
AT5G64410	OPT4	-0.46	**-3.19**	**-2.52**	**-1.53**
AT4G27730	OPT6	-1.20	**-1.71**	**-1.86**	0.12
AT1G30400	MRP1	0.10	**-0.44**	-0.13	**-0.88**
AT2G34660	MRP2	0.30	**0.94**	**1.32**	0.21
AT2G47800	MRP4	-0.22	0.24	**0.99**	0.08
AT3G62700	MRP10	0.28	**0.63**	**0.51**	**-0.35**
AT2G47730	GSTF8	**1.68**	**1.55**	**0.84**	**-1.15**
AT2G29490	GSTU1	0.21	**1.63**	**1.57**	**2.05**
AT2G29460	GSTU4	-0.42	**4.30**	**3.44**	**6.52**
AT2G29450	GSTU5	**2.40**	**3.25**	**1.65**	0.04
AT2G29440	GSTU6	0.49	**3.04**	**2.59**	**2.86**
AT1G74590	GSTU10	-0.16	-0.01	**-0.85**	-0.11
AT1G10370	GSTU17	-0.22	**0.88**	**0.90**	-0.09
AT1G78380	GSTU19	**0.54**	0.53	0.31	0.00

HL treatment was performed by exposing plants to HL for 15 (HL15) and 60 min (HL60), while recovery was performed by transferring HL60 samples to GL to recover for 15 (R15) and 60 min (R60). The genes involved in GSH metabolism were taken from (Dorion et al., 2021). GST/GSTU genes involved in GSH-

conjugation of 12-oxo-phytodienoic acid (OPDA) were taken from (Skipsey et al., 2011). The significant expression values ($p < 0.05$) are presented in bold. Gene names are listed in the text.

Supplementary Table S4: Marker genes and the primers used in the reverse transcription quantitative PCR (RT-qPCR) assay.

Gene ID	Marker Gene	Forward Primer	Reverse Primer
AT4G27960	UBC9 (Ref. gene)	TCCTACTTCATGTAGCGCAGGAC	TCCTCCAGAATAAGGGCTATCCG
AT5G24770	VSP2	GGACTTGCCCTAAAGAACGACACC	GTCGGTCTTCTCTGTTCCGTATCC
AT3G25770	AOC2	CTGCCAAGAAGAACCTCACTGC	TCTTGAACTTTGCTTGGTCTAGGG
AT1G30135	JAZ8	GGTCGGATCCTCCAAACAAGTC	TCGTCGTGAATGGTACGGTGAAG
AT5G13220	JAZ10	TCGAGAAGCGCAAGGAGAGATTAG	TCGTTTAGGCCGATGTCGGATAG
AT1G43160	Rap2.6	CCATTGATTACCGGTTCAGCTGTG	CCTCTCCAAGGACATTGAGCTTTC
AT5G42650	AOS	GGTGGCGAGGTTGTTTGTGATTG	TTCGTAACGGCGACGTACCAAC
AT2G26150	HSFA32	GTCAAGCGAGTTTGTGGAGGAC	CGGCTTCTAAACACCGTTCAGC
AT1G52560	HSP26.5	AAAGGCTCACCTGAGGAAGACG	TCATCAGGCAACGATAAGCTCGTG
AT3G46230	HSP17.4A	TGGAGGCCGAAGAACAAACGTG	TCCTTCGAACGGATCCCATACGTC
AT1G53540	HSP17.6C	TTTCGATCCGTTCTCGCTGGATG	TGCGTTTGCCAATCCTGACG
AT5G52640	HSP90.1	AAGCTCGATGGACAGCCTGAAC	TCCCAAGTTGTTCACCAAATCTGC

References

Dorion S, Ouellet JC, Rivoal J (2021) Glutathione metabolism in plants under stress: Beyond reactive oxygen species detoxification. *Metabolites* **11**: 641

Gollan PJ, Aro EM (2020) Photosynthetic signaling during high light stress and recovery: targets and dynamics. *Philosophical Transactions of the Royal Society B: Biological Sciences* **375**: 20190406

Op Den Camp RGL, Przybyla D, Ochsenbein C, Laloi C, Kim C, Danon A, Wagner D, Hideg É, Göbel C, Feussner I, Nater M, Apel K (2003) Rapid induction of distinct stress responses after the release of singlet oxygen in Arabidopsis. *Plant Cell* **15**: 2320–2332

Skipsey M, Knight KM, Brazier-Hicks M, Dixon DP, Steel PG, Edwards R (2011) Xenobiotic responsiveness of Arabidopsis thaliana to a chemical series derived from a herbicide safener. *J Biol Chem* **286**: 32268–32276

Xin Z, Zhao Y, Zheng ZL (2005) Transcriptome analysis reveals specific modulation of abscisic acid signaling by ROP10 small GTPase in Arabidopsis. *Plant Physiol* **139**: 1350–1365

Zhang N, Zhou S, Yang D, Fan Z (2020) Revealing shared and distinct genes responding to JA and SA signaling in Arabidopsis by meta-analysis. *Front Plant Sci* **11**: 908
